# Using intervention mapping to design and implement quality improvement strategies towards elimination of lymphatic filariasis in Northern Ghana

**DOI:** 10.1371/journal.pntd.0007267

**Published:** 2019-03-25

**Authors:** Alfred Kwesi Manyeh, Latifat Ibisomi, Frank Baiden, Tobias Chirwa, Rohit Ramaswamy

**Affiliations:** 1 Division of Epidemiology and Biostatistics, School of Public Health, University of the Witwatersrand, Parktown, Johannesburg, South Africa; 2 Dodowa Health Research Centre, Dodowa, Ghana; 3 Nigerian Institute of Medical Research, Lagos, Nigeria; 4 Faculty of Infectious and Tropical Diseases, London School of Hygiene and Tropical Medicine, London, United Kingdom; 5 Public Health Leadership Program, Gillings School of Global Public Health, University of North Carolina, McGavran-Greenberg Hall, Chapel Hill, North Carolina, United State of America; Task Force for Child Survival and Developmentorce for Global Health, UNITED STATES

## Abstract

**Introduction:**

The Global Strategy to Eliminate Lymphatic Filiariasis (GFELF) through Mass Drug Administration (MDA) has been implemented in Ghana since the year 2000 and transmission has been interrupted in 76 of 98 endemic districts. To improve the MDA in the remaining districts with microfilaria (MF) prevalence above the 1% threshold for the interruption of transmission, there is a need to identify and implement appropriate quality improvement (QI) strategies. This paper describes the use of intervention mapping to select QI strategies to improve an existing evidence-based MDA program in Northern Ghana.

**Methods:**

Due to the complexities associated with implementing evidence-based programs (EBP) such as the lymphatic filariasis MDA and variability in the context, an initial assessment to identify implementation bottlenecks associated with the quality of implementation of lymphatic filariasis MDA in the Bole District of Ghana was conducted using a mixed methods approach. Based on the findings of the initial assessment, a context specific QI strategy was designed and operationalized using intervention mapping strategy in terms of seven domains: actor, the action, action targets, temporality, dose, implementation outcomes addressed, and theoretical justification.

**Results:**

The initial needs assessment shows that the persistent transmission of lymphatic filariasis in the Bole District is characterized by high levels of refusal to ingest the drug, high levels of reported adverse drug reactions, low MDA coverage at community level, poor adherence to the MDA protocol and non-participants’ responsiveness.

**Conclusion:**

This study has shown that it is feasible to develop a context specific QI strategy for an existing evidence-based intervention based on an initial needs assessment through stakeholder participation using the IM approach. However, working (towards) QI requires more time than is usually available in public health service. Sufficient theoretical knowledge of implementation research and experience with technical IM experts must be available.

## Author summary

Lymphatic Filariasis is a significant health problem threatening 1.1 billion people in 55 countries globally. After 18 years of implementing MDA in Ghana, LF transmission still persist in some districts with microfilaria prevalence level high above the threshold needed for the interruption of transmission of the disease. For Ghana to meet the 2020 NTDs deadline, it is important to identify context specific need-based QI strategies to make the drug accessible to all eligible individuals in the endemic communities. Hence, this study has demonstrated the feasibility of using IM to develop and implement a context specific QI strategy for an existing evidence-based intervention (LF MDA) towards elimination of lymphatic filariasis as a public health challenge in Bole District of Northern Ghana.

## Introduction

Over 120 million people have been infected with Lymphatic Filariasis (LF) worldwide [1, 2]. This disease is caused by microscopic, thread-like worms (filarial) known as *Wuchereria bancrofti*, *Brugia malayi or B*. *timori* [1, 2]. Lymphatic Filariasis is the second leading cause of permanent disability after leprosy [3] and is considered a Neglected Tropical Disease (NTD) [4].

In 1997, the World Health Assembly passed a resolution to eliminate LF by the year 2020. The global program to eliminate the disease was then launched by the World Health Organization in 2000 [5]. Towards achieving this target, an evidence-based intervention (EBI) that involved annual mass treatment with a single-dose of diethylcarbamazine (DEC) or Ivermectin (IVM) in combination with Albendazole (ALB) was employed by endemic countries. Multiple rounds of this treatment are necessary to reduce the microfilaria prevalence to zero [6].

In June 2000, Ghana became one of the first West African countries to implement the MDA strategy through the Ghana Filariasis Elimination Program (GFEP). The target of the program in Ghana was reducing the LF prevalence level to below 1% after 4–6 rounds of high-coverage (>80%) MDA [7].

After 15 years of implementing the MDA program in Ghana, a total of 76 districts out of the initial 98 endemic districts achieved the target [8]. The remaining 22 districts still have prevalence above the 1% threshold [8] and are now referred to as LF “hotspot” districts. Bole District in Northern Ghana is one of these districts. The 2016 microfilaraemia prevalence in Bole District was 1.9% with some communities having prevalence rate as high as 5.9%. A baseline study to assess the coverage and quality of implementation of an ongoing Mass Drug Administration (MDA) in the Bole District of Northern Ghana was conducted. The baseline results described in detail later in this paper indicated that, the persistent transmission of the lymphatic filariasis in Bole District is characterized by high levels of refusal to ingest the drug, high levels of reported adverse drug reactions, low MDA coverage at a community level, poor adherence to the MDA protocol and a lack of responsiveness by nonparticipants.

Based on the results of the baseline, a quality improvement (QI) intervention was proposed to improve the quality of implementation. Experts in the field of implementation research have emphasized the need for a rigorous, theory-based approach to select intervention strategies that link to the barriers. One such approach to selecting an evidence-based strategy is intervention mapping (IM).

Intervention mapping is a program planning framework for the development of theory and evidence-based health interventions [9]. Intervention mapping offers procedures and tools for establishing empirical and theoretical bases for selecting health intervention programs, for the translation of theory into actual intervention activities and materials, for the management of program adoption and implementation and for partnership between health system, researchers, priority groups and stakeholders [9]. It also describes the intervention development process. Intervention mapping enables the development of health interventions that may accomplish program objectives and match priority populations and intervention contexts. The IM strategy suggests that the priority population and program implementers should be involved in all stages of decision making to ensure that the intervention package is based on the needs of the community [9]. IM is also a planning approach that is grounded in evidence-based strategies, and as fundamentals for taking an ecological approach to assessing and intervening in health problems through promotion of stakeholder engagement at all levels [9]. IM has been effectively used to design, implement and evaluate interventions that showed substantial increase in application of disease prevention programs [10].

For Ghana to be able to meet the 2020 NTDs deadline, it is important to strengthen the lymphatic filariasis MDA delivery systems through context specific QI strategies to make the drug accessible to all eligible individuals in the endemic communities. In this paper, we describe how intervention mapping was used in selecting QI strategies to improve LF MDA outcomes in Bole District of Northern.

## Methods

### Ethics statement

All study participants were adults. Permission was sought from all research participants through written consent. Ethical approval was given by the Human Research Ethics Committee (medical) of the University of the Witwatersrand, Johannesburg, South Africa (Clearance Certificate No. M170219) and the Ghana Health Service Ethics Review Committee (Clearance Certificate No. GHS-ERC: 04/02/2017).

### Intervention mapping

Intervention mapping (IM) is a systematic process involving six key steps [11]. The six steps of IM process consist of 1) initial needs assessment, 2) developing program objectives, 3) selection of intervention methods and practical strategies, 4) producing program components and materials, 5) plan for adoption and implementation and, 6) designing an evaluation plan. The advantage of IM is its use of models, theories and engagement with key stakeholders to identify context specific intervention. IM is used as a planning process to advance theories and evidence-based health promoting intervention programs. In this study, an adapted context specific IM process was used. In order to develop a context specific QI intervention within an evidence-based program such as the lymphatic filariasis MDA, we started with an initial needs assessment using mixed methods approach. Based on the finding of needs assessment, we identified evidence-based and practical strategies backed by the concepts of the Global Program to Eliminate Lymphatic Filariasis and the Ghana Filariasis Elimination Program. The selection of the strategies was grounded on models, theories and evidence from other related studies. The QI strategies were developed into consolidated implementation package based on evidence and stakeholders’ brainstorming sessions. Monitoring intervention and evaluation was the final step of the IM process. The planning of the monitoring and evaluation was guided by the QI strategies. The monitoring and evaluation were done to assess both the process and impact of the QI intervention on knowledge of LF, knowledge of the MDA and the MDA coverage. The subsequent sections stipulate five steps of IM process used to design a consolidated QI implementation package for LF MDA in Northern Ghana.

Before the commencement of the study, stakeholders’ meetings were held with the managers of the NTDs program in Ghana at national, regional and district levels to gather support for the project. This preliminary work provided a foundation from which the researchers worked with existing NTD program and community leaders to develop the QI intervention.

#### Initial needs assessment

The initial assessment was conducted to identify implementation bottlenecks of the ongoing MDA. This strategy was employed to gain a deeper understanding of the critical issues affecting the implementation of the LF MDA from the perspective of the services providers (health workers) and the clients (community members).

The needs assessment involved: (a) 20 in-depth interviews with District Director of Health Service, Sub-district Heads of Health Service, Disease Control Officers and Community Drug Distributors, followed by 16 key informant interviews with Chiefs/Opinion leaders and noncompliant community members who were purposively selected, (b) surveys with 222 randomly selected community members and (c) analysis of secondary MDA coverage data of 150485 participants.

**Key informant and in-depth interviews:** Semi-structured interview guides were used for the data collection. They were translated from English to the local language before the field work began. To ensure that the versions in English were the same as those in the local languages, back-to-back translation strategy was used. The guides covered broad areas such as knowledge and understanding of LF, knowledge and understanding of the MDA process and implementation challenges of the LF MDA in the study district. All interviews were recorded using a digital voice recorder, translated and transcribed verbatim. Field notes were also taken which were turned into data documents for analysis. The transcribed data and field notes were inputted into a Microsoft Word processor and exported into NVivo Version 10 for coding. Framework method of qualitative data analysis was used [12, 13]. This method involved familiarization, identification of thematic framework, indexing, charting, mapping and interpretation [12–14]. Explore and query functions in Nvivo were used to provide a descriptive perspective of the data as per framework method of qualitative data analysis [15]. The descriptive accounts were compared with the existing literature to provide an explanation and interpretation of the data. The results were summarized based on the themes that emerged from the data with key quotes from participants.

**Survey:** The survey was conducted with 222 randomly selected community members. The sample size (n) was determined using the formula: n = (Z_α/2_)^2^ *(p*q) _**/**_ d^2^ [16]. The sample size calculation was based on the assumption that 65% [17] of the population were aware of the MDA program and ingested the drugs, Z_α/2_ is the confidence level at 95% = 1.96, p is the coverage, q is (1-p) and d is the level of precision. The participants were randomly selected using simple random sampling strategy. A random number generator was used to select the desired number of participants using the LF community registers as sampling frame. Information gathered includes socio-demographic characteristics of participants, knowledge of MDA, understanding of the MDA process, stakeholder engagement before and during the MDA, reasons for refusal to ingest the drug and knowledge of LF. Other information collected includes knowledge of how LF is acquired, knowledge of signs associated with LF and knowledge of LF prevention and misconceptions about LF. These variables were measured by correct answers to signs associated with LF, LF prevention methods and how LF is acquired. Comprehensive knowledge of LF was estimated by the ability to identify correctly at least two major ways of preventing LF transmission, mode of LF transmission, and rejecting at least two misconceptions about how LF is acquired. A descriptive analysis was carried out in Stata version 14.2 and results were presented in tables.

**Secondary MDA data:** The secondary data include information on adverse drug reaction, MDA coverage, noncompliance and non-eligibility of 176 857 participants. From the available secondary data, this information on MDA coverage, noncompliance and non-eligible of 176 857 participants was extracted.

#### Selecting evidence-based and practical strategies

Results from initial needs assessment were used to identify the possible areas of intervention. The main aim of this intervention was to improve the quality and level of coverage of the LF MDA in the study area, hence we identified evidence-based, cost effective and context specific strategies which was acceptable for all stakeholders.

The QI strategies are based on the concept of the Global Program to Eliminate Lymphatic Filariasis (GPELF) [18] and the aim of the Ghana Filariasis Elimination Program [7]. The GPELF is an innovative, public–private partnership for health improvement. The selection of the QI strategies in this study was also grounded on evidence from other LF MDA studies such as improving service delivery strategies of the MDA, effective stakeholder engagement, and improving compliance at the level of the community and the individual level [7, 18, 19].

The success of LF MDA is shown to be backed by community sensitization and mobilization through evidence-based, multi-channel communication approaches with key messages on treatment and side effects combined with a high visibility if the MDA should be pioneered [17–23].

As evident in a study in Nepal by Adhikari et al, awareness of MDA campaigns had significantly increased the level of compliance among participants. Individuals who had knowledge of side effects during MDA campaigns had a lower prevalence of non-compliance [24].

Community leaders were involved, based on the World Health Organization’s (WHO) recommendation that community involvement in health programs will not only help to remove the burden of dependency that characterizes health development work but also creates general awareness among local people about the need for their involvement in all forms of health development [25].

Evidence elsewhere shows that the involvement of local leaders, religious and political authorities in the MDA activities enhances the participation, coverage and compliance of MDAs [18, 20, 21, 26].

A study conducted in 2017 in Southern Ghana shows that knowledge, attitude and practices relating to LF and the MDA improved after a health education intervention [7]. It has been suggested by Njomo et al [27] that for the LF elimination program to succeed, there is a need for aggressive community sensitization towards the disease and elaborate social mobilization [21]. Thus, bridging the knowledge gap with an appropriate educational intervention is cardinal to change perceptions and popularly held-beliefs which have no scientific basis.

The selection of the QI strategies also combined several complementary theories such as, the health belief model, social cognitive theory, the community empowerment model and the social networks and social support model. These theories were used as a guide in developing the interventions package. This is because the desired change is at both the provider and the individual levels. Hence, there is the need convincing providers and the endemic community to consider the LF drug as a viable intervention option to stop transmission of LF. These theories have been applied in similar public health interventions [11, 28–30]. A practical description of the evidence-based strategies for this QI intervention were based on a model adopted from Proctor et al 2013 [31].

#### Developing the QI strategies

This stage of the IM process involved stakeholder engagement meetings. The purpose of the stakeholder discussion was for the dissemination of the initial needs assessment results and to map out tailored, discrete, need and evidence-based implementation strategies to address the specific contextual bottlenecks identified in the initial needs assessment.

The stakeholders included the District Director of Health Services, the District Health Promotion Officer, the District Disease Control Officer, the District Public Health Officer and Sub-district heads of health services from Bole District. Others involved were sub-district disease control officers, NTD focal persons, local government representatives and journalists from two radio stations.

The stakeholders were placed into seven groups to brainstorm the barriers outlined in Table 1. They were tasked with coming up with ideas that would translate into an implementation strategy to overcome the barriers identified during the needs assessment stage.

**Table 1:** Socio-demographic information of survey participants.

Variables	Frequency	Percentage
**Sex**		
Male	106	47.75
Female	116	52.25
Mean Age (SD*)	43.90 (16.57)	
**Marital status**		
Married	173	77.93
Not married	49	22.07
**Occupation**		
Farmer	158	71.17
Trader	27	12.16
Public servant	5	2.25
Unemployed	15	6.76
Artisans	17	7.66
**Religion**		
Christian	98	44.14
Moslem	100	45.05
Traditional	24	10.81
**Level of education **		
No Education	158	71.17
Primary	23	10.36
Junior High Sch.	27	12.16
Senior High Sch. and tertiary	14	6.31

The initial needs assessment shows that the persistent transmission of lymphatic filariasis in the Bole District is characterized by refusal to ingest the drug due to the fear of adverse drug reactions, poor knowledge and misconceptions of the disease, reported adverse drug reactions, poor community mobilization and sensitization, non-adherence to the direct observed treatment strategy, poor adherence to the MDA protocol and non-participants’ responsiveness as detailed in S1–S6 Tables. The barriers and needs identified are summarized in Table 2.

The ideas from the seven groups (shown in Table 2) were compiled into a consolidated QI implementation package as described in Table 4 using an implementation specifying model adopted from Proctor et at 2013. The consolidated QI implementation package is also informed by evidence and theories identified in selecting evidence-based and practical strategies. Staff of the Bole District Health Management, Sub-district Heads of Health Service and opinion and community leadership agreed that the consolidated QI package had the potential to improve the coverage and quality of implementation of the ongoing LF MDA.

**Table 2:** Barriers to the lymphatic filariasis mass drug administration in the bole district of ghana.

	Issues/Concerns	Barriers
**1**	Knowledge of LF and MDA among Drug Distributors	• Drug distributors do not have enough knowledge of the Disease and the purpose of the MDA. Drug distributors do not understand the MDA inclusion criteria. • Drug distributors are unable to explain the importance of the MDA and the need for all to participate to the endemic communities.
**2**	Social mobilization and sensitization	• The endemic communities have little or inadequate information about the disease and the MDA. No mobilization and sensitization is done in some endemic communities.
**3**	Drug distribution process	• The duration of the MDA process is not enough to cover temporarily absent people. There is not enough time to prepare for the MDA.
**4**	Adverse drug reactions	• Community members have no knowledge about the drug reactions and what to do when they experience adverse drug reactions. Due to the low knowledge of and understanding of possible drug reactions, some community members refuse to take the drugs
**5**	Non-complaint community members	• Refusal to ingest the drug by some community people. Misconceptions about the disease by community members.
**6**	Non-adherence to the DOT strategy	• Some drug distributors do not adhere to the directly observed treatment (DOT) strategy.
**7**	Non-involvement of community leaders and other stakeholders	• Community leaders such as assembly men and women, opinion leaders and traditional leaders are not involved in the MDA exercise.

#### Monitoring and intervention evaluation

Monitoring and evaluation of the QI intervention is the final Step of IM. This step of the IM allows for sympathetic formative evaluation to assess both the process and impact of the intervention and whether changes need to be made [32, 33]. The monitoring and evaluation step was planned to assess the activities before, and during the implementation of the QI strategies based on the planned consolidated QI implementation package. As part of the monitoring and to ensure successful implementation of the QI strategies, a QI implementation team with representation from the district and each sub-district was formed. The general responsibility of the QI team was to ensure the smooth execution and sustainability of the QI strategies. A WhatsApp group was created to link members of the QI team. This social media platform was used to facilitate the sharing of ideas and challenges encountered during the implementation of the QI strategies. The WhatsApp platform also allowed the principal investigator to review progress, troubleshoot problems and reinforce compliance to the QI strategies during the implementation process. The sub-districts heads of health services were also task to submit a weekly written implementation report. The evaluation includes collection of qualitative (key informant and in-depth interviews) data on MDA activities before and during the implementation of the intervention, knowledge of LF, and Knowledge on MDA. The Participants of the evaluation qualitative study include District Director of Health Service, Sub-district Heads of Health Service, Disease Control Officers and Community Drug Distributors, and Chiefs/Opinion leaders. Secondary quantitative data on MDA coverage, noncompliant, non-eligible and adverse drug reaction was extracted from the database of the Bole District Health Administration from 70591 and 79894 individuals for the pre and post intervention phases of study respectively.

## Results

The study adopted intervention mapping strategy to come up with QI approaches for implementation in an existing EBP (LF-MDA program). An initial stakeholder engagement meeting involving officials of the NTDs program at National, Regional and District levels was organized during the formative stages of the study. The IM process comprised an initial needs assessment study, stakeholder engagement discussions in a systematic process grounded on models, theories and evidence from other related studies.

As shown in Table 1, more than half (52.25%) of the survey participants were female. The mean age of the participants is 43.90 years (16.57 standard deviation). Majority of the participants (77.93%) were married. In terms of occupation, 71.17% of the respondents were farmers and 45.05% were Moslems. While greater proportion of the survey participants (71.17%) had no education, only 6.31% had senior high school and tertiary levels of education.

The results of the brainstorming session (based on the results of the needs assessment) are presented in Table 3. The seven stakeholder groups suggested solutions ranging from the training of drug distributors (CDDs), community education, proper supervision, health education and community feedback, to the involvement of opinion/religious and community leaders. The selection of the final consolidated QI strategies was based on available evidence from literature as shown under the justification domain in Table 4.

**Table 3:** Suggested solutions from stakeholder brainstorming sessions.

Issues/Concerns	Challenges	Suggested solutions by District Health Management Team	Suggested solutions by Tinga sub-district	Suggested solutions by the assembly (local government) participants	Suggested solutions by Bamboi sub-district	Suggested solutions by Mandari sub-district	Suggested solutions by Bole sub-district	Suggested solutions by Mankuma sub-district
1. Training of drug distributors	• Drug distributors do not have enough knowledge of the disease and the purpose of the MDA. Drug distributors do not understand the MDA inclusion criteria. Drug distributors are not able to explain the importance of the MDA and the need for all to participate.	Distributors should be trained on causes of disease, its mode of transmission, signs and symptoms and ways of prevention. Exclusion criteria (pregnant women, breastfeeding mothers, seriously sick people and under height people) should be made known to distributors.	• CDDs should be given educated in MDA and the disease. Adequate education of the exclusion criteria should be given to CDDs. CDDs should be well trained on the importance of the MDA to ensure community people participate. CDDs should be trained in each community.	• In-service training should be organized for drug distributors in the district.	• Proper education about the disease which includes the causes, signs and symptoms, mode of transmission and preventive measures. • Educate the drug distributor on eligibility and non-eligibility by listing the criteria and providing hard copies for the CDDs to refer to when in the field. • CDDs should be educated on the importance and benefits of taking the drugs.	• Adequate training of drug distributors (all inclusive). Hold training sessions for drug distributors at different locations • CDDs should be literate people. Training should include practicals.	• Days for training should be increased to two days. Training decentralization. Increase the number of CDDs.	• Drug distributors should be educated on the disease and the purpose of the MDA. Education on the MDA inclusion criteria. Selected CDDs should be trusted and also have knowledge and the capability to convince the community of the MDA program.
2. Social mobilization and sensitization	• The endemic communities have little or inadequate information of the disease and the MDA. No mobilization and sensitization is carried out in some endemic communities.	• Include stakeholders (Chiefs, assembly members, etc) in the MDA exercise. Organize community durbars and meet social groups to explain MDA relevance. Make announcements in churches, mosques, schools, etc.	• CDDs should be trained to deliver messages to the community people through regular gatherings. Health education should be carried out at CWC, OPD and ANC levels before and after the MDA.	• Community durbars should be organized in the endemic communities to educate the people before administering the drugs.	• Hold meetings with stake holders eg. chiefs, Imams, Pastors, elders, etc. to educate them on the disease, the MDA activities and when it would begin. Dissemination of information to the community members by door to door education, holding durbars/ outreach sessions at market squares, churches, mosques, community groups, school health programs and during ante-natal and post-natal sessions.	• Intensify social mobilization through home visits, outreaches, durbars, school health programs, statics (OPD, CWC, ANC)	• Home visitsSchool health serviceOutreach serviceDurbarsOPD level service	• Organize durbars to include major stakeholders (Chiefs, assembly members, religious leaders, etc.) Mobilization and sensitization should be done through gong-gong beating, church services, mosque sessions, youth meeting and groups (JAKSALLY Group) OPD, outreach.
3. Drug distribution process	• The duration of the MDA process is not sufficient to account for temporarily absent people. There is inadequate time to prepare for the MDA.	• 3 weeks of preparation before the distribution of drugs are required. One-month period is required for re-visits before submitting reports to region.	• Duration of MDA should be extended in order to accommodate revisits and reduce absenteeism. MDA period should be changed from the rainy season to the dry season.	• A follow up visit should be carried out after the mass distribution.	• Appeal for extension of MDA period to meet the demands of the people. Appeal for the MDA activities to be carried out during the dry season. Group proposed early notification for proper preparation.	• Maintain a yearly calendar on MDA. The training of drug distributors should be in the dry season.	• Extend the duration of the MDA exercise. Follow up visits. Provide a second opportunity for MDA.	• Duration of MDA should be extended to cater for absent people. Revisits by CDDs to places where there were absent people, located by markingPre-information should be given to communities in order to make time for the MDA.
4. Adverse drug reactions	• Community members have no knowledge of possible drug reactions and what to do if they experience adverse drug reactions. Due to limited knowledge of possible drug reactions, some community members refuse to take the drugs.	• Sensitize community members on the adverse effects of the drugInform participants on where and when to go for help if they experience any reactions. Let participants know that the treatment of reactions would be catered for by the health system.	• CDDs should be trained to explain the side effects of the drug to the people and also to inform them to report to the nearest health facility in the case of any adverse reactions to the drug.	• Drug distributors should educate community members on some of the reactions and what to do in the case of such reactions.	• Educate people on the adverse effects of the drug. Advise them to report to the nearest Health Facility when experiencing any reactions from the drugs. Conduct follow up visits.	• Education of drug distributors on some of the perceived drug reactions and how to address them. Education of drug distributors on the side effects and what to do.	• Health education on early identification and early reporting of drug reactions. Health education on side effects.	• Community members should be well informed about the adverse effects of the drug and report to the nearest heath facility or the CDD in the case of any reactions. Community members should be well informed about the importance of the drugs and be assured of the availability of the health staff’s support.
5. Non-complaint community members	• Refusal to ingest the drug by some individuals. Misconceptions about the disease by community members.	• Educate community members on the importance of taking the drugsEducate them on the myths and known misconceptions held by the community.	• Stakeholders should be involved during the MDA in order to explain the merits of the drug. Interpersonal relationships during the MDA.	• Community members should be educated on the importance of taking the drug.	• Health education on the side effects of the drug and its management. Awareness creation during social mobilization through durbars, at churches, mosques, etc.	• Proper education of community members on the benefits of the drugs and the dangers of not taking them. • Educate and encourage some community members to be part of the sensitization process.	• Health education on the importance of the drugs and possible side effects. Good interpersonal relationships.	• CDDs should observe the in-take of the drug by community members during the MDA. They should be educated about the causative agent of the disease and that it is not caused by witchcraft, in-take of oily foods and neither is it hereditary.
6. Feedback to communities	• No feedback is given to the endemic community on the MDA and the status of the disease prevalence.	• Feedback would be provided to the stakeholders (Chiefs, Imams, pastors, assembly members, health staff, teachers, etc).	• Feedback should be given to stakeholders after the MDA.	• There should be feedback to the endemic communities in the form of ranking.	• Organize durbars, school health programs and group meetings to disseminate the feedback of the disease prevalence to the community members.	• Organize durbars, school health and community outreaches with key messages on Challenges, successes, support and importance of the MDA.	• DurbarsReview meetingsDuring MDA exercise.	• CDDs and supervisors should be educated on giving feedback to the communities and the importance of this feedback.
7. Non-adherence to the DOT strategy	• Some drug distributors do not adhere to the DOT strategy.	• Explain the essence of DOTs to the CDDs and encourage them to be patient with community members.	• CDDs should ensure that drugs are taken in their presence. Supervision of CDDs during MDA.	• Drug distributors must adhere to the DOT strategy and encourage community members to comply.	• Educating the volunteers on the DOT strategy during the training of CDDs.	• Drug distributors should be well educated on DOT and strictly comply with the requirements. Drugs should not be given to a third party.	• Supervision of CDDs.	• CDDs should be made to understand that most people do not take the drugs, hence the need to observe the community members taking the drug. CDDs should desist from the I don’t care attitude.
8. Non-involvement of community leaders and other stakeholders	• Community leaders such as assembly men and women, opinion leaders and traditional leaders are not involved in the MDA exercise.	All stakeholders would be involved before, during and after the exercise.	• Stakeholders should be involved in the MDA exercise.	• Opinion leaders in the communities should be actively involved in the MDA exercise.	• Hold stakeholders meeting with the chiefs, elders, religious leaders, traditional rulers, etc. focusing on the MDA exercise.	• Proper community entry–To hold meetings with key stakeholders and community leaders to explain the purpose of the exercise and seek their approval and support. Community leaders should be made to understand that they are part of the successes and the challenges. Always plan with the community.	• Involve leaders and other stakeholders in the MDA training	• Community leaders and stakeholders should be actively involved in the organization of the MDA exercise.

**Table 4:** Description of consolidated implementation package.

Domain*	Strategy: Training of Community Drug Distributors (CDDs)	Strategy: Social Mobilization and Sensitization	Strategy: Involvement of Community Leaders and other Stakeholders	Strategy: Drug Distribution Process
**Actor(s)**	• District Director, Sub-district NTD focal persons, disease control officers and implementation/QI team (District and sub-district NTDs focal persons and heads, DCO, HPO, Assemblymen/women and PI) who have been trained.	• Intervention implementation/QI team (District and sub-district NTDs focal persons, opinion, religious and traditional leaders and PI).	• Intervention implementation/QI team.	• Intervention implementation/QI team and CDDs
**Action(s)**	• Train drug distributors to have a good knowledge and to be able to explain the importance of the MDA exercise to community members. Train drug distributors to be able to convince every qualified person in the endemic area to participate in the MDA exercise. Train drug distributors to know the inclusion and exclusion criteria of the MDA. Train drug distributors to know the possible adverse drug reactions and explain them to the community members. The drug distributors should be taught the skills of communication and interaction and the importance of being patient and tolerant with difficult community members. Strong enforcement of the MDA procedures, in particular, DOT policy during training and supervision.	• An evidence-based, multi-channel communication strategy to result in high levels of awareness among community members, (radio discussions and announcements, announcements in churches, mosques, schools, etc., community durbars and meetings with social groups to explain MDA relevance and public/community announcements). Focus key messages on cause and mode of transmission of the disease, importance of the MDA and how to identify, what to do and minimize adverse drug reactions.	• Community/Opinion leaders such as Chiefs, Assembly men/women, religious and traditional leaders should be involved in the MDA exercise.	• An adequate number of days should be dedicated to the distribution exercise (not less than 1week) The distribution should reach people in institutions, markets places, offices and homes. People with higher level qualifications and a good knowledge of the MDA should be sent to institutions and offices to distribute the drug. Enough days should be assigned to the MDA activities. Strong enforcement of the DOT policy.
**Target (s) of the action**	• Drug distributors in the endemic communities.	• People in the endemic communities.	• Community leaders.	• People in the endemic communities.
**Temporality**	• The drug distribution should start within one week after the training of drug distributors.	• Social mobilization and sensitization should start two weeks before and should continue during the MDA.	• Before, during and after MDA exercise.	• During the drug distribution
**Dose**	• The training of the CCDs should be detailed enough to equip them well for the MDA task and the training period should not be more than one day to enhance their active participation in the training.	• Each endemic community should have at least two social mobilization and sensitization exercises (community durbar, school education, information centre announcement, education at church and mosque, and or radio talk shows etc.) for the start of MDA and at least one during MDA.	• Every endemic community must have a community leader representing it.	• The distribution exercise should not be less than 1 week in the endemic district. • Over 80% of the people in the endemic communities must be covered.
**Implementation outcome (s) and effect**	• Increase the level of adherence to LF MDA implementation procedures and participants’ responsiveness.	• At least 15% increase in MDA coverage and reduction in the number of refusals (increased participant responsiveness).	• At least 15% increase in MDA coverage and reduction in the number of refusals (increased participant responsiveness).	• At least 15% increase in MDA coverage and reduction in the number of refusals (increased participant responsiveness).
**Justification**	• Researchers suggest that drug distributors are the interface between MDA programs and their targeted population hence their adequate training is crucial to the success of the MDA [18, 20, 34, 35].	• It has been shown that evidence-based, context specific and multi-channel social mobilization and sensitization is required for the LF elimination program to succeed [7, 20, 21].	• Stakeholder engagement and involvement in LF MDA cannot be overemphasized [7, 18, 20].	• The MDA implementation process is very important for participants’ responsiveness to the program [7, 20].

Note CDD: Community Drug Distributors, DCO: Disease Control Officer, HPO: Health Promotion Officer, QI: Quality Improvement, DOT: Direct Observed Treatment MDA: Mass Drug Administration. *Model adopted from Proctor et al 201

The stakeholder engagement meeting for dissemination of baseline results and IM towards the intervention phase of the study was held in the district capital of Bole District. The purpose of the stakeholders’ engagement was to disseminate the findings of the baseline study and to map out tailored, discrete, need-based implementation strategies to address the specific contextual bottlenecks identified in the preliminary study in order to improve the fidelity of the MDA and its coverage.

Table 4 presents the consolidated QI strategies that was implemented. The researchers together with the stakeholders came up with the final cost effective and evidence-based QI strategies based on the model adopted from Proctor et al (2013) [31]. These strategies were informed by theories [11, 28–30], the concept of the GPELF [18] and evidence from other related studies [17–23, 26]. The components of the implemented strategies include training of community-based drug distributors (CDDs), social mobilization and sensitization, involvement of community leaders and other stakeholders and the drug distribution process. Each of these strategies have seven (7) domains namely; actors, actions, target of action, temporality, dose, implementation outcome effects and justification.

### The domains

#### The actor

The actors used in this context are stakeholders who will deliver the QI strategies. The actors for this study include District and Sub-districts NTD focal persons, disease control officers, health promotion officers, local government representatives (assemblymen/women) and the Principal Investigator (PI).

#### The action

The actions are the specific processes or steps and activities to be undertaken in a specified sequence. For this study, the actions for each of the four strategies are specified in Table 4. These actions include training activities, multi-channel communication strategies, involvement of community leaders and increase in duration of MDA activities for wider coverage of eligible population.

#### Targets of actions

An implementation strategy is a function of where it is directed to have impact. Hence, it is important to specify the target because it helps focus the strategy and suggests where and how an outcome of interest should be measured. The targets of actions in this study were community drug distributors, people in the endemic district and community and opinion leaders. A total of 162 community drug distributors where trained.

#### Temporality

The order or sequence in which a QI strategy is implemented is very important in the execution process of the strategy. This study specified when each strategy should be used as shown in Table 4.

#### Dose

These are details about the intensity of the implementation strategies required to have the needed effect. This ranges from number and duration of training sessions, number of social mobilization and sensitization activities per community for the duration of the drug distribution exercise. The training was for one day, but the number of social mobilization and sensitization activities varies from community to community.

#### Implementation outcomes and effect

Implementation outcomes are a purposive and deliberate actions or activities to implement new strategies or practices. Implementation outcomes serve as indicators of the implementation success, act as proximal indicators of implementation processes and key intermediate outcomes [36]. Implementation outcomes therefore serve as necessary preconditions for achieving desired changes or results [37]. The implementation outcome in this study is increasing adherence to the LF MDA implementation process and involvement of the endemic community. Resulting in increasing the MDA coverage by 15% and reducing the number of refusals as shown in Table 4.

#### Justification

The selection of the implementation strategies in this study is based on a proactive needs assessment of barriers or factors affecting the implementation of the LF MDA in the study district. The strategies are also supported by current literature as shown in Table 4 and programmatic rationale through stakeholder engagement in the brainstorming exercise which is deemed appropriate to address the specific bottleneck posed by the implementation context.

The results of the secondary data as detailed in S1 Table shows that, the total MDA coverage for pre-intervention is 82.03% while that of the post intervention is 83.40%. The reported pre-intervention adverse drug reaction is 0.13% while that of post intervention is 0.02%. (See S1 Table for detail results). While the average community and sub-district MDA coverage in the pre-intervention is 82.67% and 81.32% respectively, that of post-intervention is 81.50% and 87.80% respectively as shown in Table 5.

**Table 5:** Summary statistics of lymphatic filariasis mass drug administration coverage in Bole.

Summary Statistics	Pre-intervention, 2016		Post Intervention, 2017	
	Community Coverage	Sub-district Coverage	Community Coverage	Sub-district Coverage
Mean	82.67	81.32	81.50	87.8
Standard Deviation (sd)	12.19	14	11	8.9
Range	47.52–100	70.00–98.90	58.5–100	79.8–99.00

There seems to be improvement in the knowledge about the disease and understanding of the MDA among the study participants and in the study, area as shown in the qualitative results shown in S7 Table.

## Discussion

This paper has used the problem of persistently-high prevalence of LF in the Bole district of Ghana to demonstrate the step-by-step procedure for selecting and tailoring QI strategies to an evidence-based intervention program. The QI strategies take into account local contextual needs through needs assessment and stakeholders’ engagement [38]. The IM method used in this study has galvanized stakeholders around common goals and generate consensus regarding improving an ongoing evidence-based intervention at a low-cost as shown in other studies [38, 39]. The process emphasizes stakeholder contribution in coming up with a QI strategy [38, 40].

As suggested in other studies [38, 41], this article provides a clear description of the implementation strategies used as detailed in Table 4. In line with the article by Proctor et al (2013) [31], this article carefully names, conceptually defines, operationalizes strategies and specifies them according to features such as (1) the actor, (2) the action, (3) action target, (4) temporality, (5) dose, (6) implementation outcome [42] and (7) the empirical, theoretical, or pragmatic justification for use of the strategy [31].

In order to show a clear link between the strategies and the bottlenecks that they are intended to address, an IM process and Proctor et al (2013) implementation specifying model was used to clarify how and why carefully selected and tailored implementation strategy is proposed to work as suggested by other researchers [31, 38].

Improving the quality of evidence-based interventions to achieve expected results has proven to be a challenging task for most health system programs [43–51]. Studies [49–51] suggest that barriers and facilitators must be taken into consideration and that different strategies may be required to overcome challenges associated with interventions, implementation contexts, and stages of implementation [38]. The intervention package of the current study is in line with this suggestion. The processes presented in this article have increase the rigor of selecting and tailoring an appropriate QI strategy as demonstrated by Powell et al (2015) [38]. This article also provides a step-by-step procedure for selecting and tailoring QI strategies and for engaging stakeholders who are very important in the success of the intervention. It further shows the links between strategies and barriers and other contextual factors.

Describing, implementing, and sustaining any evidence-based intervention is a complex undertaking [52, 53]. This is because implementing quality improvement strategies (1) are usually multi-component and (2) must be adapted to local contexts. The local contexts in which these interventions are implemented are themselves complex because of multiple interacting levels with wide variation from one setting to another [52, 54]. Hence, implementation research requires trans-disciplinary expertise which are not routinely part of the health system in Ghana. The emerging implementation research provides a systematic set of principles, methods and expertise to improve the quality and effectiveness of evidence-based interventions within the health system [52].

The move toward improving evidence-based health system programs with a more thoughtful and systematic application of implementation research will enhance the expansion and legitimacy of the field of implementation research. The scientific rigor of proposed QI studies, strengthening evidence-based interventions to achieve expected results and providing support for implementation of QI strategies will be enhanced.

The demonstration of this study was to provide a systematic method for selecting and tailoring QI strategies of evidence-based interventions to contextual needs in collaboration with stakeholders. This will provide an understanding of how, when, where, and why implementation strategies are effective in integrating evidence-based interventions and improving clinical outcomes in the health sector [55].

### Limitations

Although the insight provided by this paper will be valuable to other low-resource settings seeking to use evidence-based QI strategies to improvement the quality of the lymphatic filariasis mass drug administration towards the elimination of the disease as a public health problem, it is important to note that, the QI strategies could not incorporate other issues such as incentives/motivation (monetary rewards) for CDDs, transportation related challenges and the timing of the MDA. These were part of the challenges identified during the initial needs assessment. These are decisions that could only be made at regional and national levels of neglected tropical diseases program due to the cost implications.

### Conclusion

Although the overall MDA coverage in the study district increased from 82.03% in pre-intervention to 83.40% in the post-intervention, the social mobilization strategies such as (community durbar, gong-gong beating and announcements in churches and mosques) has not been effective in peri-urban and urban communities. In designing a QI intervention within a district, there is the need to consider sub-district and community level dynamics. The “one-size-fits-all” intervention does not work even within the same district.

The use of radio stations, information vans and posters for social mobilization and sensitization could have been more effective in peri-urban and urban communities as shown other studies [7, 56, 57]. Due to the financial implications, these methods of social mobilization sensitization (which the funding of this study could not support) were not included in the consolidated intervention package implemented.

The timing of the MDA which coincide with the farming season and Muslim fasting period leads to missing of eligible people and refusal to ingest the drug respectively.

For Ghana to meet the 2020 NTDs deadline, it is important to strengthen the lymphatic filariasis MDA delivery system through context specific QI strategies to make the drug accessible to all qualified individuals in the endemic communities. Hence, there is the need for concerted efforts to improve the quality of implementation and coverage of the ongoing lymphatic filariasis MDA campaigns in the Bole District of Northern Ghana.

This study has shown that it is feasible to develop a context specific QI strategy for an existing evidence-based intervention based on an initial needs assessment through stakeholder participation using IM approach. However, working according to QI requires more time than is usually available in public health services. Furthermore, sufficient theoretical knowledge of implementation research and experience with technical IM aspects has to be available.

## Supporting information

S1 Table Pre and post intervention mass drug administration coverage in Bole Districts. (DOCX)

S2 Table Knowledge about lymphatic filariasis in Bole District. (DOCX)

S3 Table Knowledge of mass drug administration in Bole District. (DOCX)

S4 Table Exploring knowledge and understanding of lymphatic Filariasis. (DOCX)

S5 Table Exploring the understanding of mass drug administration exercise in study districts. (DOCX)

S6 Table Exploring suggestion on how to reach community members and improve mass drug administration. (DOCX)

S7 Table Post intervention knowledge about the disease and mass drug administration. (DOCX)
