# Supplementary material for: Using intervention mapping to design and implement quality improvement strategies towards elimination of lymphatic filariasis in Northern Ghana
Source: PLoS Negl Trop Dis. 2019 Mar 25;13(3):e0007267. doi: 10.1371/journal.pntd.0007267 (PMC6448919; doi:10.1371/journal.pntd.0007267)
Supplement: S1 Table — (DOCX) [file pntd.0007267.s001.docx]

Supporting information

**Table S 1: Pre and post intervention mass drug administration coverage in Bole Districts.**

| **Pre-intervention** | | | | |
| --- | --- | --- | --- | --- |
| **Characteristics** | **Compliant* (%)** | **Noncompliant**** | **Non-eligible*** (%)** | **Total**  **(%)** |
|  |  | (%) |  |  |
| **Sex** |  |  |  |  |
| Male | 30273  (80.40) | 5018  (13.32) | 2364  (6.27) | **37655**  **(100)** |
| Female | 27635  (83.91) | 1739  (5.28) | 3562  (10.81) | **32936**  **(100)** |
|  |  |  |  |  |
| **Total** | **57908**  **(82.03)** | **6757**  **(9.58)** | **5926**  **(8.39)** | **70591**  **(100)** |
|  |  |  |  |  |
| Adverse Reaction | 180 (0.31) † |  |  |  |
|  |  |  |  |  |
| **Post-intervention** | | | | |
| **Sex** |  |  |  |  |
| Male | 34391  (78.36) | 7131  (16.25) | 2366  (5.39) | **43888**  **(100)** |
| Female | 32232  (89.52) | 1468  (4.08) | 2306  (6.40) | **36006**  **(100)** |
|  |  |  |  |  |
| **Total** | **66623**  **(83.40)** | **8599**  **(10.76)** | **4672**  **(5.85)** | **79894**  **(100)** |
|  |  |  |  |  |
| Adverse Reaction | 16  (0.02) † |  |  |  |
|  |  |  |  |  |

**Compliant are those who ingested the drug. **Noncompliant is made up of people who refused to ingest the drug and those absent at the time of the MDA. ***Non-eligible is made up of Pregnant, Breastfeeding mothers, seriously sick, under-height children (details can be found in* S1 Table*).* †*This is per the number of people who ingested the drug*.
