# Supplementary material for: Using intervention mapping to design and implement quality improvement strategies towards elimination of lymphatic filariasis in Northern Ghana
Source: PLoS Negl Trop Dis. 2019 Mar 25;13(3):e0007267. doi: 10.1371/journal.pntd.0007267 (PMC6448919; doi:10.1371/journal.pntd.0007267)
Supplement: S3 Table — (DOCX) [file pntd.0007267.s003.docx]

Supporting information

**Table S 3: Knowledge of mass drug administration in Bole District**

|  |  | |
| --- | --- | --- |
| **Variables** | **Frequency** | **Percentage** |
| **Awareness of LF MDA** |  |  |
| Yes | 102 | 45.95 |
| No | 120 | 54.05 |
| **Source of MDA information** |  |  |
| Radio | 2 | 2.25 |
| Health workers | 6 | 5.41 |
| Posters | 0 | 0.00 |
| Family members | 7 | 6.76 |
| Church/mosque | 18 | 18.02 |
| Community volunteers | 37 | 36.49 |
| Gong-gong | 14 | 13.51 |
| Neighbors/friends | 18 | 17.57 |
| **Was there any public education before MDA** |  |  |
| Yes | 40 | 39.64 |
| No | 51 | 50.00 |
| Don’t know | 11 | 10.36 |
| **Have you ever taken the LF drug** |  |  |
| Yes | 98 | 44.14 |
| No | 82 | 36.94 |
| Can't remember | 42 | 18.92 |
| **How was the drug administered** |  |  |
| Direct observation treatment | 140 | 63.06 |
| Given to beneficiary to take at his/her convenience | 82 | 36.94 |
| **Why some community members’ refusal to ingest drug** |  |  |
| Fear of side effects | 143 | 64.41 |
| Level of knowledge of the disease | 20 | 9.01 |
| Don’t think will get the disease | 17 | 7.66 |
| Think only sick people should take | 15 | 6.76 |
| Too many drugs | 18 | 8.11 |
| Religious beliefs/superstition | 6 | 2.70 |
| Taking other medication | 2 | 0.90 |
| Have taken the drugs for too many times | 1 | 0.45 |
