# Supplementary material for: Using intervention mapping to design and implement quality improvement strategies towards elimination of lymphatic filariasis in Northern Ghana
Source: PLoS Negl Trop Dis. 2019 Mar 25;13(3):e0007267. doi: 10.1371/journal.pntd.0007267 (PMC6448919; doi:10.1371/journal.pntd.0007267)
Supplement: S4 Table — (DOCX) [file pntd.0007267.s004.docx]

Supporting information

**Table S 4: Exploring Knowledge and Understanding of Lymphatic Filariasis**

| **Knowledge on causes of the disease** | | |
| --- | --- | --- |
| **Reference** | **Respondent** | **Quote** |
| Quote KC1 | CDD | “...the disease is caused by insect bite and worms in our system preventing the free flow of blood to our joints causing the swollen body parts” |
| Quote KC2 | Health worker | “what I know is there is an insect that causes that [...] filariasis is caused by a fly [...] the flies are common in communities along the rivers banks” |
| Quote KC3 | Noncompliant | “…there is a hole in this community when you step in it, you get LF” |
| Quote KC4 | Opinion leader | “[...] it is a result of curse [...] so if a person steal something they are curse with the disease.” |
| **Signs and symptoms** | | |
| Quote SS1 | Noncompliant | “[…] the leg swells with rashes all over it.” |
| Quote SS2 | CDD | “...swollen leg [...] when you yourself sees the person you will just know that this is LF. It also starts by itching before it eventually develops into that big thing” |
| Quote SS3 | Opinion leader | “What I know is sudden swelling of some parts of the body, especially the legs” |
| **Prevention and treatment measures** | | |
| Quote PT1 | CDD | “The disease can be prevented by sleeping under mosquito net, intake of LF drugs and regular check up in the hospital can all prevent LF infections” |
| Quote PT2 | Opinion leader | “[...] by taking the LF drugs we have been receiving [...] So those that refuses to take the drug wouldn’t be able to prevent the disease but we those that take the drug automatically prevents our system from getting LF” |
| Quote PT3 | Health worker | “The disease can be prevented through the Ivermectin and Albendazole [...], surgeries can be performed on the legs but I don’t think our people can afford that” |
