# Supplementary material for: Using intervention mapping to design and implement quality improvement strategies towards elimination of lymphatic filariasis in Northern Ghana
Source: PLoS Negl Trop Dis. 2019 Mar 25;13(3):e0007267. doi: 10.1371/journal.pntd.0007267 (PMC6448919; doi:10.1371/journal.pntd.0007267)
Supplement: S5 Table — (DOCX) [file pntd.0007267.s005.docx]

Supporting information

**Table S 5: Exploring the Understanding of Mass Drug Administration Exercise in Study Districts**

| **Timing and Understanding of MDA** | | |
| --- | --- | --- |
| **Reference** | **Respondent** | **Quote** |
| Quote UN2 | Health worker | “The purpose is to eliminate the LF disease in the system. It gives them the immunity to fight the disease when they take they drugs” |
| Quote TM1 | CDD | “[...] the last distribution that we did occurs during the rainy season which is not the best as most people goes to farm” |
| Quote TM2 | Health worker | “June and July are the periods normally used for the distribution of the drugs which is the beginning of the farming season here” |
| Quote TM3 | Opinion leader | “The exercise is done in the beginning of the farming season [...] the kind of work (farming) the people do here is a challenge as most of them go to sleep in their farm during the farming season [...] |
| **Activities before MDA** | | |
| Quote AM1 | CDD | “Before the distribution of the drugs, I make announcement at the Mosque, and information centers to inform the people about the date the medicine will be distributed.” |
| Quote AM2 | Health worker | “Before the MDA exercise we were trained at Bole and we also trained the volunteers here in Tinga. The drugs and other materials were sent to us after the training” |
| Quote AM3 | Health worker | “[…] we first go for training at the district level then we come to train at the sub-district level [...] then to the community to give health information to community members before the actual day of distribution” |
| Quote AM4 | CDD | “Most often, it is the hospital that informs me as a volunteer and I will also let them make announcement through gong-gong beating. Also, it is me the volunteer who will move from house-to-house to inform the people again about the drug and its purpose before they will accept and take the drugs” |
| Quote AM5 | CDD | “When the time is due for us to start distributing the LF drug, they call us from Bole to come for training and collection of the drug. When we get home, we inform the town crier to announce to the community members. We also make announcements in the church because the community is a Christian dominated community. We tell them when the exercise is going to begin and we ask that they stay at home for this exercise but it will interest you to know that they refuse and go to their usual work places and come back in the evening. This usually does not help the work at all” |
| Quote AM6 | CDD | “We the community volunteers are always invited to training at Bole District Hospital, where we are given a refresher training on the drug distribution, after which the date and duration of the drug distribution exercise is set for each community. We the volunteers upon reaching the community, inform the chief of the impending exercise. The chief then make the town crier or the gong-gong beater to announce to the people of the exercise and the date of commencement. After this is done, we start the drug distribution” |
| Quote TD3 | CDD | “[...] we were trained on LF and how to take peoples height, administer the drugs to the people [...] Also, how to register births and pregnant women” |
| **Activities During the MDA** | | |
| Quote AD1 | CDD | “I start by planning my movement and how to identify homes that have not been completely administered and those that are completed. I do this by numbering the various homes before entering and circle it if completely administered, but I leave it open if I have some household members missing or absent and I have to return to that house to attend to them. I also decide on what time is best and appropriate to get the people at home to reduce the amount of movement to and backward for absent people. I also get myself some indelible ink which I put on a finger of those who have taken the drug to prevent over-dose, after taking their heights and giving them the drugs.” |
| Quote AD2 | Noncompliant | “[...] they take peoples’ height before the drugs are given but they do tell us that one cannot take it when he drinks alcohol” |
| Quote AD3 | Opinion leader | “They use the stick to take your measurement before giving you the drugs” |
| Quote AD4 | CDD | “MDA exercise is carried out using the register in each household together with stick for height measurement [...]” |
| Quote AD5 | Health worker | “Children who are under-height, pregnant women and seriously sick persons are not given the drug. But aside these groups of persons, every other person takes it” |
| **Adherence to DOT** | | |
| Quote AO1 | CDD | “I leave it [the drug] with those who insist on taking it at their convenience” |
| Quote AO2 | CDD | “since everybody in the community need the drug and I know most of the people, if someone is not at home at the time of the distribution I normally leave his own[drug] with someone in the house for him” |
| Quote AO3 | CDD | “Most of them collect the drug from you telling you that they will take it at their convenience, but it was later revealed that most of these people do not take the drug after all” |
| Quote AO4 | Health worker | “I always make sure all qualify person in the house are given the drug to ingest in the presence of the CDD before they mark it in the register.” |
| **Perception and side effect of the drug** | | |
| Quote PS1 | CDD | “They usually complain and sometimes attack me [...] because of the reactions or side effects they have after taking the drug [...] I try to explain or convince them but at times it doesn’t work.” |
| Quote PS2 | Opinion leader | “Some people told me that their house people don’t take medicine so they are not ready for these drugs also. Others also have the beliefs that the drugs are meant to kill people gradually in other to reduce the population growth. Some are with the perception that the drugs are family planning drugs” |
| Quote PS3 | Health worker | “[...] they say they were vomiting, and feeling dizzy, but sometimes most of them don’t eat before taking the drug. But we have not recorded any serious side effect that should scare somebody not to take the drug” |
| Quote PS4 | Opinion leader | “people complained about swollen testicles and some people too say when they take it [the drug] they have some itches in their body [...] some people also say their they have bodily pain when the ingest the drug [...]” |
| **Challenges faced by CCDs and health workers** | | |
| Quote MF1 | Health worker | “We don’t have motor bikes; fuel is number two and some of the motor bikes breaks down and means to go and bring them is another problem. Flooding of roads to some communities is also a big problem here. |
| Quote MF2 | CDD | “I will start with the issue of motivation; when I started as a volunteer, I was given recognition each time I visited the clinic. I was also treated for free and my family too. But it all ended when they introduced the national health insurance system. I sometime work on empty stomach which is a challenge to my work” |
| Quote MF3 | CDD | “Poor knowledge of the MDA program is a big challenge. Transportation difficulty is also another challenge because some of the communities are Fulani settlements and they are far from town. Motivation is poor, as we sometimes work on empty stomachs.” |
| Quote MF4 | Health worker | “Those that go to work early and it is hard to get them during MDA exercise in this community. Fueling the motorbike to revisit those who were absent during my first visit is something that worries me.” |
| **Reasons for refusals** | | |
| Quote RR1 | Health worker | “[...] some people say it is a disguised family planning drug.” |
| Quote RR2 | Opinion leader | “The people who normally refuse to take the drug are those who had bad experience after taking it or hear others say bad thigs about the drug [...]they are scared that they might not be able to go about their economic activities due to effects of the drug.” |
| Quote RR3 | CDD | “[...]but most people refuse most often to take it [drug] because they believe when they take it some parts of their bodies get swollen. Others say they get itching skins.” |
| Quote RR4 | Noncompliant | “Yes, one man took the drugs and fall sick and died that is why these community members refuse to take the drugs” |
| Quote RR5 | Opinion leader | “The side effects and the size of the drugs prevent some people from taking it especially” |
| Quote RR6 | Noncompliant | “The last time I took the medicine I had lots of side effects […] I was vomiting, itching and had rashes on my body […] so I stopped taking it. Due to this problem and I know many people who do not want to take it due to the same problem.” |
| Quote RR7 | Health Worker | “They complain of side effects and that when they take it, they can’t do any work again until after some number of days. They say they become very weak and sick” |
| Quote RR8 | CDD | “[...] yes, at times, some people refuses to take the drug of fear of side effect and some other reasons but when I bring in the assembly man and we explain to them there agree to participate.” |
| Quote RR9 | CDD | “There is no problem reaching people with the drugs except the effect of the drug side effect that makes some people to refuse taking the drugs” |
| Quote RR10 | Noncompliant | “There was a time the drug distribution was ongoing and a woman came from ‘Sawla’ to a wedding in this community and also took the drugs. After taking the drug all her body was swollen and we had to rush her to hospital in Bole and that is the reason why some of us don’t want to take the drugs” |
| Quote RR11 | CDD | “[...] people are still finding it difficult to understand and accept the need to take the drugs. The side effects and lack of education on the part of the health officials is affecting its intake” |
| Quote RR12 | Opinion Leader | “[…] some people in this community, especially the young ones (youth), stop taking the medicine just because they think they don’t have the disease. I think they are influenced by others because they don’t even any reaction when they take the drugs but they have stopped.” |
| Quote RR13 | Noncompliant | “They are supposed give those with LF but this community they give everybody. But is those who are suffering from LF that are supposed to take the drugs” |
| Quote RR15 | Health worker | “[...] members of some churches said their church doesn’t take it [the drug] [...] some other people too have their own personal beliefs [...] a man said I won’t take it because I can’t stop taking alcohol but they are isolated cases” |
