# Supplementary material for: Using intervention mapping to design and implement quality improvement strategies towards elimination of lymphatic filariasis in Northern Ghana
Source: PLoS Negl Trop Dis. 2019 Mar 25;13(3):e0007267. doi: 10.1371/journal.pntd.0007267 (PMC6448919; doi:10.1371/journal.pntd.0007267)
Supplement: S6 Table — (DOCX) [file pntd.0007267.s006.docx]

Supporting information

**Table S 6: Exploring Suggestion on How to Reach Community Members and Improve Mass Drug Administration.**

| **Reference** | **Respondent** | **Quote** |
| --- | --- | --- |
| **Suggestions on how to reach community members** | | |
| Quote SC1 | Noncompliant | “the hospital/clinic should always create awareness through the radio and other means for any drug distribution. With this, the people can even trace me to my house in case I couldn’t reach them” |
| Quote SC2 | Opinion Leader | “Massive and frequent education about the exercise as well as its benefits, and also provide means of transport to the volunteers” |
| Quote SC3 | CDD | “They should give me motor bike, increase volunteer allowance and mass education of community members” |
| Quote SC4 | Health worker | “Those eligible as in the case of adult males who take so much alcohol in this community and as such refuse to take the drug because of the alcohol, must be strategically taken care of, so that they can take their alcohol and still take the drug. Otherwise, we cannot get all of them to take the drug because they will never skip taking alcohol” |
| Quote SC5 | Health worker | “The involvement of the community leaders during the MDA is very important. Their involvement make our work very easy.” |
| Quote SC6 | CDD | “...they mostly prefer the drug delivered to them in the evening, especially the men. This is because most of them take alcohol and they believe that when they get it in the evening, they can take it before going to bed” |
| Quote SC7 | CDD | “Well, I think we should be supported with either a bicycle or motorbike to assist us with the movement in and around the community, as some of the places are far from each other; - I also think that the issue of motivation must be given a closer look” |
| Quote SC8 | CDD | “They should always be public education before the drug distribution. This will make it easier for me when I am doing the drug distribution” |
| **Suggestions on how to improve MDA** | | |
| Quote SI1 | CDD | “More education should be done before the distribution so that one can even take it [the drug] when she/he is sick without any fear” |
| Quote SI2 | Opinion leader | “...improvement on the work of MDA depends on the education on the part of the health officials to community members on the importance of taking the drugs” |
| Quote SI3 | Noncompliant | “They should add bed nets when they are coming to distribute the drugs, it will encourage people to take the drugs. This our community members find it difficult to believe issues so if they could find time to call them for a meeting and educate them on that like it will also help” |
| Quote SI4 | Noncompliant | “They should reduce the quantity of drugs they give; they give plenty and ask you to take all” |
| Quote SI5 | CDD | “The chief must be pre-informed so that he can also organize his community members and the surrounding villages to create awareness about the exercise. Also, provision of means of transport to the volunteers” |
| Quote SI6 | CDD | “I agree that as a volunteer I am not to being paid [...] considering the recent responsibilities conferred on us, which do not allow us to be frequent on our farms but always at home doing one activity to assist our communities, I suggest that certain incentives, especially money should be given to us to enable us hire labour to weed our farm lands for us to also be able to get some food stuffs for our families at the end of the farming season.” |
| Quote SI7 | Health Worker | “I think, there is the need to intensify sensitization to educate the people more about the safety and possible side effects of the drugs [...] The community need to understand that the side effects are normal and will not harm them […] They should also be made to understand the importance of taking the drugs” |
