# Supplementary material for: Using intervention mapping to design and implement quality improvement strategies towards elimination of lymphatic filariasis in Northern Ghana
Source: PLoS Negl Trop Dis. 2019 Mar 25;13(3):e0007267. doi: 10.1371/journal.pntd.0007267 (PMC6448919; doi:10.1371/journal.pntd.0007267)
Supplement: S7 Table — (DOCX) [file pntd.0007267.s007.docx]

Supporting information

**Table S 7: Post intervention knowledge about the disease and mass drug administration**

| **Knowledge on causes of the disease** | | |
| --- | --- | --- |
| **Reference** | **Respondent** | **Quote** |
| Quote KD1 | *Opinion leader* | *“…it is a disease that affects the human body and allows some fungi to grow on the affected part and at a point you will see the affected area growing to an abnormal position. Sometimes if it is the foot you will see that foot extraordinary fatter than the other foot, if it the hand too, the same thing. But the most people I have seen being affected by this disease have it on the foot and hardly have I seen somebody being affected by the arm.”* |
| Quote KD2 | CDD | *“It is a disease that when a mosquito bites a person who is not immunized and comes to bite you then you also become infected.”* |
| Quote KD3 | CDD | *“…it is a disease that affects the lymph system which is mostly caused by the mosquito. Mostly when it does happen it is characterized by swelling and the body parts it affects are the extremities and the Scutum and other parts of the body.”* |
| Quote KD4 | CDD | *“We can talk about preventing ourselves from getting bitten by the mosquito, which sleeping under nets can be one. Wearing protective clothing can also be one for those who go to the farms.”* |
| **Broad understanding of the MDA** | | |
| Quote UD1 | CDD | *“We do the exercise because we don’t want anyone to get the disease in addition to those who already have it.”* |
| Quote UD2 | Health worker | *‘The distribution of the drug is an attempt to eradicate the disease totally from this area.”* |
| Quote UD3 | Opinion leader | *“...it has to do with distribution of medicine to people in our communities so that they are protected from getting the disease and mostly it happens once in a year... Trained volunteers go from house to house to distribute the tablets to everyone.”* |
| Quote UD4 | Health worker | *“We do what we call social mobilization and we get the stakeholders involve. We have meeting with the stakeholders and we discuss what we about to do and we let them know the reason for carrying the exercise because without the stakeholders we can’t get the community to do the exercise...we meet with them then the information is sent out... at the stakeholders meetings, we have the volunteers involve then afterwards we orientate the volunteers on what to do... then we send information across to enforce the information within the community.”* |
| Quote UD5 | Opinion leader | *“The last time we were informed about the exercise, we beat the ‘gong-gong’ in the community to inform community members of the exercise and when it would take place. We then advise them to make sure they avail themselves to take the medication. We also advice and encourage them to stay away from alcohol on the day of the exercise in order to avoid any complications.”* |
| **Differences between pre and post intervention MDAs** | | |
| Quote DM1 | CDD | *“Formerly they will say they don’t know you but now they say you have not come to my house to give me the medicine.”* |
| Quote DM2 | Opinion leader | *“One major improvement is that we were given the assurance that if anyone faces any adverse effect after taking the drugs they can report at the health facility and they would be treated for free and this really boosted the confidence of the people in the drugs.”* |
| Quote DM3 | CDD | *“As I said earlier, the compliance of the people was very high in the last exercise...those who used to complain of reactions to the drug have now seen the benefits because we always convince them that the reactions to the drugs they experience are due to the fact that the organisms that cause the disease is already in their body and so the drug is working on it therefore, they need to continue taking the drugs to completely kill the organisms...previously, people used to reject the drug and will never take it no matter what you say or do.”* |
| Quote DM4 | Health worker | *“The compliance was better now because they understood the exercise and so they were willing to take the drugs... sometimes those who were absent even trace me to come for theirs when they return.”* |
| Quote DM5 | Health worker | *“They were not willing to swallow the drug previously but last year they were willing due to the announcements and community education before the distribution.”* |
